# Cyclooxygenase-2 blockade can improve efficacy of VEGF-targeting drugs

**DOI:** 10.18632/oncotarget.3437

**Published:** 2015-01-31

**Authors:** Isabel Ben-Batalla, Miguel Cubas-Cordova, Florian Udonta, Mark Wroblewski, Jonas S. Waizenegger, Melanie Janning, Stefanie Sawall, Victoria Gensch, Lin Zhao, Iñigo Martinez-Zubiaurre, Kristoffer Riecken, Boris Fehse, Klaus Pantel, Carsten Bokemeyer, Sonja Loges

**Affiliations:** ^1^ Department of Hematology and Oncology, BMT with Section of Pneumology, Hubertus Wald Tumorzentrum, University Comprehensive Cancer Center Hamburg, University Medical Center Hamburg-Eppendorf, Hamburg, Germany; ^2^ Department of Tumor Biology, Center of Experimental Medicine, University Medical Center Hamburg-Eppendorf, Hamburg, Germany; ^3^ Department of Clinical Medicine, The Arctic University of Norway, Tromsø, Norway; ^4^ Research Department Cell and Gene Therapy, Clinic for Stem Cell Transplantation, University Cancer Center Hamburg, University Medical Center Hamburg-Eppendorf, Hamburg, Germany

**Keywords:** breast cancer, anti-angiogenic therapies, Cox-2, CAFs

## Abstract

Anti-angiogenic therapies were approved for different cancers. However, significant primary and secondary resistance hampers efficacy in several tumor types including breast cancer. Thus, we need to develop clinically applicable strategies to enhance efficacy of anti-angiogenic drugs.

We report that anti-angiogenic therapies can induce upregulation of cyclooxygenase-2 (Cox-2) and of its product prostaglandin E2 (PGE_2_) in breast cancer models. Upon Cox-2 inhibition PGE_2_ levels were normalized and efficacy of anti-vascular endothelial growth factor receptor 2 (anti-VEGFR-2) antibodies and sunitinib was enhanced. Interestingly, both treatments exerted additive anti-angiogenic effects. Following Cox-2 inhibition, we observed reduced infiltration of tumors with cancer-associated fibroblasts (CAFs) and lower levels of pro-angiogenic factors active besides the VEGF axis including hepatocyte growth factor (HGF) and basic fibroblast growth factor (FGF2). Mechanistic studies indicated that Cox-2 inhibition reduced PGE_2_-induced migration and proliferation of CAFs via inhibiting phosphorylation of Akt.

Hence, Cox-2 inhibition can increase efficacy of anti-angiogenic treatments and our findings might pave the road for clinical investigations of concomitant blockade of Cox-2 and VEGF-signaling.

## INTRODUCTION

Tumor angiogenesis represents an important hallmark of cancer [1, 2]. Thus, significant efforts were made in the past 20 years to develop monoclonal antibodies and small molecule tyrosine kinase inhibitors (TKIs), which are mainly targeting the VEGF pathway because this pathway was considered indispensable for tumor neovascularization [3-5]. These VEGF (receptor) inhibitors (VEGF(R)Is) include bevacizumab, aflibercept, sunitinib, pazopanib and sorafenib and were approved for the treatment of different cancers either as single agents or in combination with standard chemotherapy [5-7]. Bevacizumab showed clinical efficacy in various advanced common cancers including colorectal cancer, lung cancer, ovarian cancer and renal cancer [5, 7, 8]. In metastatic breast cancer, bevacizumab was approved in 2004, but this approval was revoked in 2011 in the USA due to relatively low clinical activity at the expense of significant side effects [9]. VEGF-directed TKIs have proven largely ineffective in breast cancer [10]. Interestingly, ramucirumab, a monoclonal antibody targeting VEGFR-2 represents a promising new anti-angiogenic agent because it was recently shown to prolong survival in gastric cancer as a single agent as well as in combination with paclitaxel, a tumor type in which other anti-angiogenic drugs had failed so far [11]. However, in patients with metastatic breast cancer a large phase III trial with chemotherapy plus ramucirumab did not meet its endpoint [12]. Since breast cancer represents the most common cause of cancer-related death in women and seems rather resistant against different anti-angiogenic drugs, novel strategies to improve the efficacy of anti-angiogenic therapy in breast cancer resistance are urgently warranted. The possibility to increase efficacy of anti-angiogenic drugs at lower than maximum tolerated dose (MTD) levels of anti-angiogenic drugs would also be desirable, because their side effects often require dose reductions in cancer patients [13].

Efficacy of VEGF(R)Is is limited by complex primary and evasive resistance mechanisms many of which are mediated by tumor-infiltrating stroma cells [6, 14, 15]. Within the diverse tumor stroma, CAFs and myeloid cells including myeloid-derived suppressor cells (MDSCs) and tumor-associated macrophages (TAMs) are key players mediating anti-angiogenic therapy resistance [6, 14-16]. Mechanistically, stroma cells can mediate resistance by secreting pro-angiogenic or pro-lymphangiogenic mediators besides the VEGF-axis and by suppressing the host anti-tumor immune response amongst other mechanisms [17].

Anti-angiogenic therapies elicit hypoxia in different pre-clinical cancer models, which is considered an important driving force of stroma-mediated resistance [18-20]. Thus, hypoxia-induced targets may be useful to overcome anti-angiogenic therapy resistance. Cyclooxygenase-2 (Cox-2) levels are upregulated in hypoxic conditions [21, 22] and can induce angiogenesis via mechanisms distinct from the VEGF axis [23]. It was demonstrated that different Cox-2 inhibitors reduce tumorigenesis and tumor progression. For instance, celecoxib significantly decreased the incidence of mammary tumors in MMTV/neu mice [24] and also lowered metastasis to the lung in a murine mammary cancer model [25]. In addition SC-236 and indomethacin inhibited angiogenesis by reducing VEGF levels in 4T1 tumors [26]. Therefore, we hypothesized that Cox-2 inhibitors might be useful to increase efficacy of anti-angiogenic drugs in experimental breast cancer by targeting a pro-angiogenic pathway distinct from VEGF inhibitors.

## RESULTS

### Anti-angiogenic therapies increase Cox-2 expression and PGE_2_ levels in breast cancer

In a first step, we wished to investigate whether anti-angiogenic therapies modulate Cox-2 expression in experimental breast cancer. In order to model available treatments, we chose anti-mouse VEGFR-2 antibodies (DC101) and the pan-VEGFR inhibitor sunitinib.

**Figure 1:**
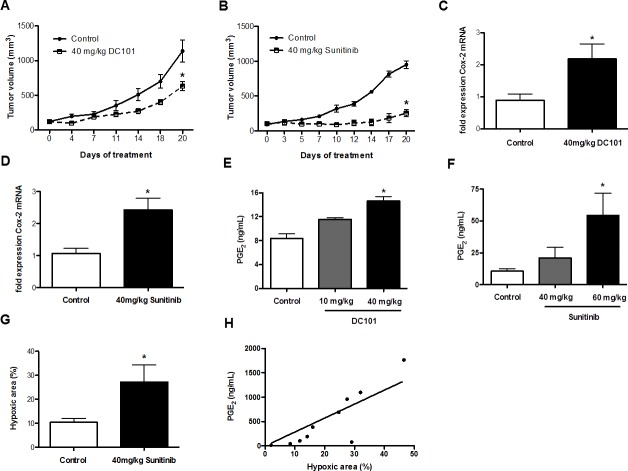
Anti-angiogenic therapies increase Cox-2 mRNA expression level and PGE production A and B, inhibition of 4T1 tumor growth by DC101 (A) or sunitinib (B) compared to control-treated mice after 20 days of treatment (n = 5; *P < 0.05, *P < 0.0001, respectively. *P* values are calculated by two-way ANOVA). C and D, qRT-PCR analysis of Cox-2 mRNA expression in 4T1 tumor tissue, showing upregulation of Cox-2 in mice treated with DC101 (C) or sunitinib (D) compared to control-treated mice (Ct values normalized to GAPDH) (n = 5; *P < 0.05, *P = 0.01, respectively). E and F, increase of PGE_2_ levels measured by ELISA in 4T1 tumors from DC101- (E) or sunitinib-treated mice (F) compared to control-treated mice (n = 5; *P < 0.005, *P = 0.03, respectively). G, Quantification of hypoxic area (in %) in 4T1 tumor sections from sunitinib-treated mice compared to control-treated mice showing a significant increase in the hypoxic area upon 40 mg/kg sunitinib treatment (n = 5; *P < 0.05). H, correlation between the hypoxic area and PGE_2_ levels in 4T1 tumors treated with 60 mg/kg of sunitinib (n = 10, r = 0.81, *P = 0.058).

We analyzed mRNA expression levels of Cox-2 in GFP^+^ tumor cells, which were FACS-sorted from end-stage 4T1 tumors after treatment with DC101 or with sunitinib (Figure 1A and B). These experiments revealed that Cox-2 mRNA was upregulated 2.5 and 2.3-fold, respectively. This upregulation occurred at standard dose levels of 40 - 60 mg/kg sunitinib [27, 28], while it was not present or not significant at lower dose levels of 10 - 20 mg/kg (Figure 1C and D and data not shown). In order to analyze whether increased expression of Cox-2 had functional consequences we subsequently determined prostaglandin (PGE_2_) levels and found that they were enhanced 1.7-fold in 4T1 tumors after anti-angiogenic therapy with 40 mg/kg DC101 and 5.2-fold after treatment with 60 mg/kg sunitinib (Figure 1E and F). As Cox-2 can be induced by hypoxia [29] we subsequently quantified hypoxia in control-treated and tumors treated with 60 mg/kg sunitinib. Consistent with published, data anti-angiogenic therapy led to an increase in intra-tumoral hypoxia. The induction of hypoxia was not significantly different between 40 mg/kg sunitinib and 40 mg/kg DC101 (Figure 1G and data not shown). Interestingly, PGE_2_ levels correlated with hypoxia (r = 0.81, p = 0.0058) (Figure 1G and H). These data indicate that treatment-induced hypoxia could be responsible for increased intratumoral PGE_2_ levels. Altogether, anti-angiogenic therapies can induce expression of Cox-2 and PGE_2_ in breast cancer at standard dose levels.

### Cox-2 inhibition decreases breast cancer growth as monotherapy and exerts additive effects in combination with anti-angiogenic therapies

**Figure 2:**
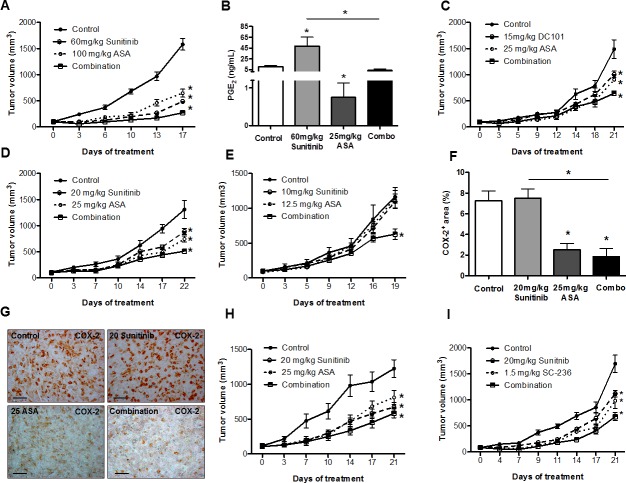
Cox-2 inhibition increases efficacy of anti-angiogenic therapies at different dose-levels in breast cancer models A and C, tumor growth curves of 4T1 tumor-bearing mice showing inhibition of tumor growth with sunitinib alone (A), and an additive effect on the tumor size reduction after combined treatment with ASA (A and C) (n = 7; *P < 0.0001. *P* values are calculated by two-way ANOVA). B, determination of the intratumoral PGE_2_ levels by ELISA reveals an increase upon therapy with sunitinib and a normalization by concomitant ASA treatment (n = 7; *P < 0.03). C, tumor growth curve showing the inhibition upon DC101 treatment (n = 7; *P < 0.0001. *P* values are calculated by two-way ANOVA) (C). D and E, the combination of intermediate (20 mg/kg) (D) or low doses (10 mg/kg) (E) of sunitinib with ASA induces an additive effect on the tumor growth inhibition in 4T1 tumor-bearing mice (n = 7; *P < 0.0001, *P < 0.0001, respectively. *P* values are calculated by two-way ANOVA). F and G, quantification of Cox-2 protein in 4T1 tumor sections (F) and representative pictures (scale bar: 50 μm) (G), indicating a reduction of Cox-2 expression after treatment with ASA and no change upon treatment with intermediate-dose sunitinib (n = 7; *P < 0.0005). H and I, tumor growth curves showing the efficacy of single treatments and an additive effect of ASA and sunitinib in 66cl4 tumor-bearing mice (n = 7, *P = 0.01, *P* value is calculated by two-way ANOVA). (H), and in 4T1 tumor-bearing mice treated with sunitinib and the specific Cox-2 inhibitor SC-236 (n = 7, *P = 0.0001, *P* value is calculated by two-way ANOVA) (I).

We hypothesized that (enhanced) Cox-2 expression could decrease the response of breast cancer to anti-angiogenic therapies and thus concomitant blockade of Cox-2 would increase their efficacy. Therefore, we treated 4T1 tumor-bearing mice with 60 mg/kg sunitinib alone and in combination with the pan-Cox inhibitor acetylsalicylic acid (ASA). We found that single treatment with ASA or angiogenesis inhibitors inhibited tumor growth and that combined inhibition of Cox-2 and VEGF(R) signaling exerted additive therapeutic efficacy at standard dose levels (Figure 2A). Consistently, intratumoral PGE_2_ levels, which were increased upon anti-angiogenic therapy, could be normalized by concomitant ASA treatment (Figure 2B).

Interestingly, also the combination of intermediate (20 mg/kg) and low doses (10 mg/kg) of sunitinib or DC101 (15 mg/kg) and ASA showed additive effects (Figure 2C-E). A comparison between the tumor growth kinetics revealed that there was no significant difference between 40 mg/kg sunitinib and 20 mg/kg sunitinib combined with 25 mg/kg ASA (Supplementary Figure S5).

At these dose levels of sunitinib or DC101 we did not observe induction of Cox-2 and PGE_2_ (see previous section), thus the mechanism underlying the additive effect of both treatments is most likely different between high and low doses of anti-angiogenic therapies. As ASA can induce downregulation of Cox-2 gene expression [30] we hypothesized that reduced Cox-2 expression levels could mediate additive anti-tumor effects of low/intermediate doses of sunitinib and ASA. Indeed, morphometric quantification of Cox-2 protein in tumor sections indicated a reduction of Cox-2 expression upon treatment with ASA, while its expression was unchanged upon treatment with 20 mg/kg sunitinib (Figure 2F and G). These data are consistent with the observed reduction of PGE_2_ levels upon treatment with ASA monotherapy (Figure 2B).

In order to rule out that additive effects of Cox-2 inhibition and anti-angiogenic therapy only occur in a single breast cancer model and might therefore not be representative we treated 66cl4 tumor-bearing mice with ASA and sunitinib. Similar to our observations in the 4T1 model we found efficacy of single treatments and an additive effect of ASA and anti-angiogenic treatment (Figure 2H). Thus, the ability of ASA to enhance efficacy of anti-angiogenic treatment is present in different breast cancer models.

Next, we wished to determine whether the observed effect is specifically due to Cox-2 inhibition and therefore treated 4T1 tumor-bearing mice with sunitinib and the specific Cox-2 inhibitor SC-236. In these experiments we also observed an additive effect of combined Cox-2 inhibition and anti-angiogenic therapy (Figure 2I). In conclusion, pan-Cox or specific Cox-2 inhibition increases efficacy of anti-angiogenic therapy at different dose-levels in different breast cancer models. For further analyses of the tumor phenotype we chose intermediate doses of anti-angiogenic agents (20 mg/kg) and ASA (25 mg/kg).

### Cox-2 inhibition and anti-angiogenic therapies exert additive anti-angiogenic effects

**Figure 3:**
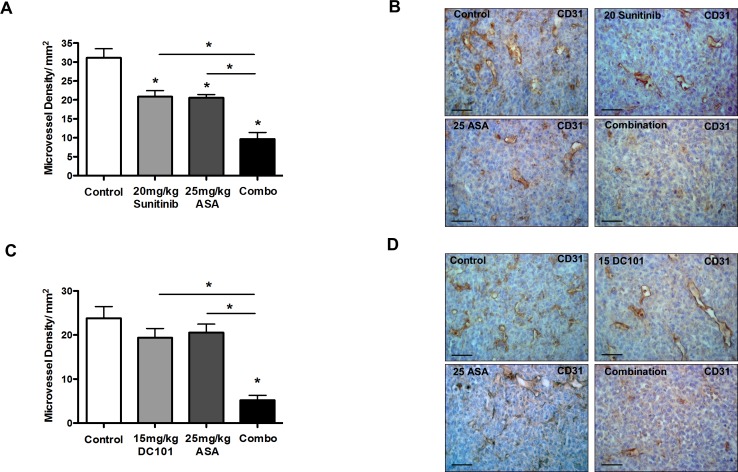
Cox-2 inhibition and anti-angiogenic therapies exert additive anti-angiogenic effects A-D, morphometric analysis of microvessel density in CD31-stained 4T1 tumor sections, indicating that the density of CD31^+^ vessels in 4T1 tumors treated with a combination of sunitinib and ASA is lower compared to the respective monotherapy (n = 7; *P < 0.003) (A and B), and the same effect is observed when DC101 is combined with ASA (n = 7; *P < 0.0001) (C and D). Representative pictures of CD31-stained tumor sections (scale bar: 50 μm) (B and D).

Previous data indicate that inhibition of Cox-2 can exert additive anti-angiogenic effects independent from VEGF signaling [23]. Based on these data we hypothesized that inhibition of Cox-2 and VEGF signaling could elicit additive anti-angiogenic effects. Indeed, the density of CD31^+^ vessels in 4T1 tumors treated with combined Cox-2 and VEGF blockade was lower compared to the respective monotherapy (Figure 3A-D). Hence, the additive anti-angiogenic effect of VEGF pathway– and Cox-2 blockade most likely represents a mechanism underlying the enhanced anti-tumor activity of both treatments.

Next we wished to investigate whether treatment with ASA influences important (pro-angiogenic) cellular components of the tumor microenvironment in order to elucidate additional mechanisms underlying the anti-angiogenic effect of ASA.

### Cox-2 inhibition and anti-angiogenic therapies reduce tumor infiltration with (activated) cancer-associated fibroblasts

**Figure 4:**
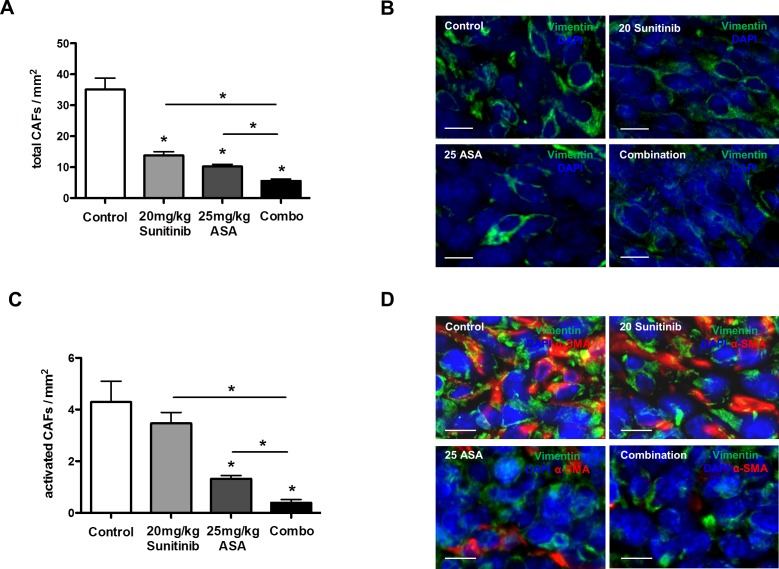
Cox-2 inhibition reduces tumor infiltration with activated cancer-associated fibroblasts A-D, histomorphometric analyses of vimentin^+^ total CAFs (A and B) and vimentin^+^α-Sma^+^ activated CAFs (C and D) in 4T1 tumor sections. Data indicate a reduction of total CAF infiltration with sunitinib and ASA monotherapy and an additive effect with the combination (n = 7; *P < 0.0001) (A and B). Quantification of vimentin^+^α-Sma^+^ CAFs reveals a reduction in the fraction of activated CAFs upon ASA treatment (n = 7; *P < 0.001) (C and D). Representative immunofluorescence pictures stained for vimentin (green), α-Sma (red) and DAPI in 4T1 tumor tissues (scale bar: 10 μm) (B and D).

Cancer-associated fibroblasts promote tumor angiogenesis by secreting a plethora of pro-angiogenic mediators, which can not be inhibited by VEGF pathway blockers [31, 32] and can therefore be considered as key stromal element mediating resistance to these agents. It is known that Cox-2 inhibition can affect the proliferative state of fibroblasts *in vitro* [33]. Therefore, we hypothesized that ASA treatment could influence numbers of tumor-infiltrating CAFs in principle. Interestingly, histomorphometric analyses of total vimentin^+^ CAFs [34] in tumor tissues treated by ASA with or without anti-angiogenic agents revealed a 3.4-fold reduction of CAFs upon treatment with ASA alone. Sunitinib monotherapy also reduced CAF infiltration and both treatments in combination resulted in an additive reduction of CAFs (Figure 4A and B). In contrast, DC101 did not significantly reduce infiltration of tumors with CAFs (Supplementary Figure S1A and B). Next, we determined numbers of vimentin^+^α-SMA^+^ activated CAFs which revealed that ASA, but not sunitinib at a dose of 20 mg/kg or DC101, reduced the fraction of activated CAFs within the total population of CAFs (Figure 4C and D; Supplementary Figure S1C and D). Higher doses of sunitinib monotherapy (40 and 60 mg/kg) could reduce both tumor infiltration and activation of CAFs (Supplementary Figure S2A and B).

**Figure 5:**
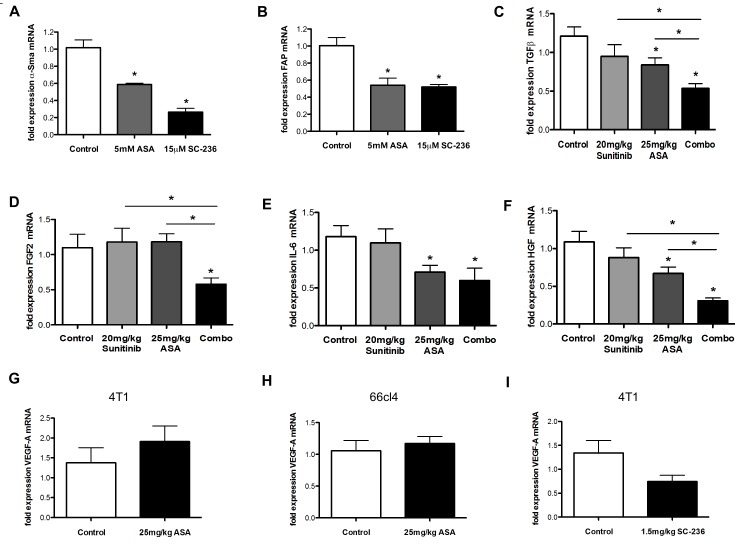
Influence of PGE and Cox-2 on the activation of CAFs *in vitro* and reduction of pro-angiogenic cytokines after ASA and sunitinib treatments *in vivo* A-I, qRT-PCR analysis of CAF activation markers α-SMA (A) and FAP (B) in CAFs isolated from tumor tissue of n=2 lung cancer patients. Showing dereased activation of CAFs by ASA or SC-236 *in vitro* (A and B) (n = 3; *P < 0.05, *P < 0.01, respectively). C-F mRNA expression levels of TGFβ (C), FGF2 (D), IL-6 (E) and HGF (F) in 4T1 tumor samples demonstrate a reduction upon combination of ASA and sunitinib treatments *in vivo* (n = 3; *P < 0.05, *P < 0.05, *P < 0.05, *P < 0.05, respectively). G-I mRNA expression levels of VEGF-A are not modified after treatment with ASA (G and H) or SC-236 (I) in 4T1 (G and I) or 66cl4 (H) tumor samples (n = 3; P = 0.426, P = 0.968, P = 0.077, respectively). Ct values normalized to GAPDH.

In order to confirm the link between Cox-2 inhibition and activation of fibroblasts we investigated the influence of Cox-2 inhibitors on the activation of CAFs isolated from tumor tissue of n=2 lung cancer patients *in vitro*. We incubated CAFs in the presence of ASA or SC-263. Subsequently, we measured the well-described CAF activation markers α-SMA and fibroblast activation protein (FAP) by qRT-PCR [35]. These experiments revealed decreased activation of CAFs in the presence of Cox-2 inhibitors (Figure 5A and B).

Thus, sunitinib and ASA exert additive effects in reducing the total number of CAFs but ASA could also decrease their activation *in vitro* and *in vivo*. DC101 did not reduce infiltration of tumor with CAFs. It is well known that activated CAFs secrete higher levels of pro-angiogenic cytokines compared to non-activated CAFs [32, 36, 37]. Thus, we hypothesized that treatment with ASA could lead to decreased levels of pro-angiogenic factors in the tumor tissue.

### Tumors treated with ASA and sunitinib contain less pro-angiogenic cytokines aside the VEGF axis

Therefore, we next determined expression levels of the following cytokines capable to promote angiogenesis in cDNA prepared from tumor tissues treated with sunitinib and/or ASA by qRT-PCR: transforming growth factor beta (TGFβ), fibroblast growth factor 1 (FGF1), basic fibroblast growth factor (FGF2), interleukin 6 (IL-6) and hepatocyte growth factor (HGF) [36-38]. Importantly, all of these mediators cannot be blocked with VEGF pathway inhibitors [39]. In addition, it was demonstrated that FGF2 mediates resistance of endothelial cells towards sunitinib [40]. Furthermore, we determined expression levels of VEGF mRNA because Cox-2 inhibitors can reduce expression of this cytokine in tumors [41, 42]. These analyses revealed that TGFβ, FGF2, IL-6 and HGF were reduced upon combination of ASA and sunitinib treatments (Figure 5C-F). Some of these mediators, including TGFβ, HGF and IL-6, were reduced by single ASA treatment and showed an additive reduction in the combination group (Figure 5C and F). VEGF mRNA expression levels were not (significantly) reduced by ASA or SC-236 (Figure 5G-I). Higher doses of sunitinib (60 mg/kg) decreased mRNA levels of FGF-2 and TGFβ while IL-6 and HGF mRNA levels were not significantly lowered (Supplementary Figure S2C-F).

Thus, sunitinib alone can decrease FGF2 and TGFβ mRNA expression levels when applied at higher concentrations while it seems to have less effects on intratumoral IL-6 and HGF mRNA expression.

Our data indicate that combined treatment with ASA and sunitinib leads to lower levels of TGFβ, FGF-2, HGF and IL-6 all of which are capable to promote angiogenesis in presence of VEGF(R)Is.

**Figure 6:**
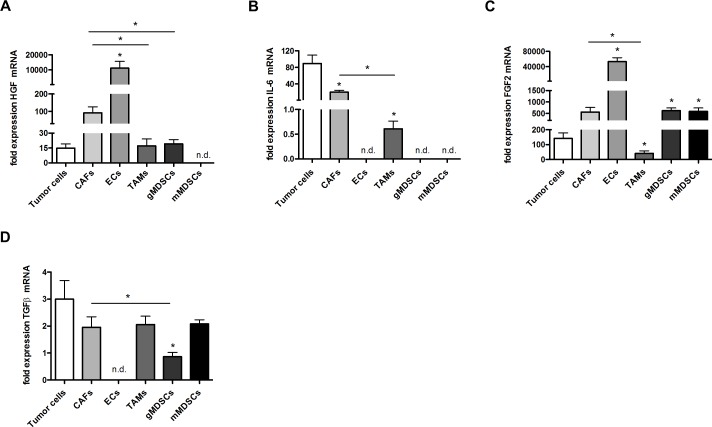
Cytokine expression in tumor and stroma cells *in vivo* A-D, qRT-PCR analysis of HGF (A), IL-6 (B), FGF2 (C) and TGFβ (D) in 4T1 different cell populations sorted from 4T1 tumors. FACS-sorted cells included tumor cells, CAFs, endothelial cells (ECs), tumor-associated macrophages (TAMs), granulocytic myeloid-derived suppressor cells (gMDSCs) and monocytic MDSCs (mMDSCs). (n = 7; *P < 0.05, *P < 0.001, *P < 0.05, *P < 0.05, respectively).

Next, we FACS-sorted important cellular constituents of untreated tumors including tumor cells, CAFs, endothelial cells (ECs), tumor-associated macrophages (TAMs), granulocytic myeloid-derived suppressor cells (gMDSCs) and monocytic MDSCs (mMDSCs) in order to analyze possible sources of these cytokines. Here, we found that HGF mRNA was predominantly expressed by CAFs and ECs while IL-6 mRNA was mainly expressed by tumor cells and by CAFs. FGF-2 and TGFβ were expressed by a wider range of cell types including TAMs and MDSCs (Figure 6A-D). Hence, the observed reductions of TGFβ, FGF2, IL-6 and HGF can not only be due to the reduction of tumor-infiltrating CAFs but also due to quantitative and/or qualitative changes in other intratumoral cell populations such as reduced numbers of ECs due to an anti-angiogenic effect of the treatments.

### Cox-2 inhibition blocks proliferation of CAFs *in vitro* and *in vivo*

Reduced numbers of tumor-infiltrating CAFs could be due to decreased proliferation, recruitment and/or migration of CAFs.

**Figure 7:**
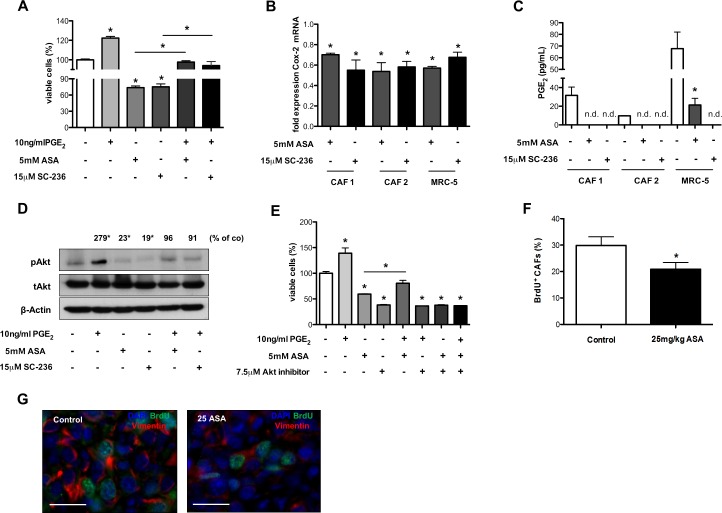
Inhibition of Cox-2 reduces proliferation of CAFs *in vitro* and *in vivo* A and E, WST-1 assay of primary CAFs showing increased numbers of CAFs after incubation with PGE_2_ which is counteracted by ASA or SC-236 treatment *in vitro* (n = 3; *P < 0.05) (A). B, qRT-PCR analysis of Cox-2 mRNA expression in primary CAFs and in the MRC-5 cell line. Results reveal reduction of Cox-2 mRNA levels upon treatment with ASA and SC-236 (Ct values normalized to GAPDH) (n = 3; *P < 0.05). C, PGE_2_ levels measured by ELISA in supernatants from CAFs isolated from tumor tissue of lung cancer patients and in the MRC-5 cell line showing a decrease of PGE_2_ secretion after treatment with ASA or SC-236 (n.d. not detectable; n = 3; *P = 0.02). D, immunoblot showing protein levels of phosphorylated Akt (pAkt), total Akt (tAkt) and β-Actin from protein extracts of primary CAFs indicating an upregulation of pAkt by PGE_2_ which is counteracted by ASA or SC-236 treatment *in vitro.* Densitometric quantification of (phosphorylated Akt/β-Actin)/(total Akt/β-Actin) (n = 3; *P < 0.01) (D). WST-1 assay showing that the Akt inhibitor (MK-2206) and ASA inhibit fibroblast proliferation alone or in combination with PGE_2_ without exerting additive effects (n = 3; *P < 0.01) (E). F and G, morphometric analyses of BrdU^+^ CAFs in 4T1 tumor sections, indicating reduced CAF proliferation upon treatment with ASA compared to control-treated tumors (n = 7; *P < 0.05) (F). Representative pictures of immunofluorescence staining for vimentin (red), BrdU (green) and DAPI (blue) in 4T1 tumor tissues (scale bar: 25 μm) (G).

First, we investigated effects of Cox-2 and PGE_2_ on proliferation of CAFs isolated from tumor tissue of lung cancer patients and of the embryonic fibroblast cell line MRC-5 *in vitro*. We incubated CAFs or MRC-5 cells with PGE_2_ and ASA or SC-263. These experiments revealed induction of CAF proliferation by PGE_2_, which was counteracted by Cox-2 inhibition (Figure 7A and data not shown). Interestingly, monotherapy with ASA or SC-263 also inhibited CAF proliferation compared to control (Figure 7A and data not shown).

Published data indicate that Cox-2 inhibitors can reduce Cox-2 levels in tumor cells leading to decreased secretion of PGE_2_ and thereby influencing the proliferative state of the cells [43]. qRT-PCRs revealed that Cox-2 mRNA levels were reduced in CAFs upon treatment with Cox-2 inhibitors (Figure 7B). Consistently, ELISAs performed with cell culture supernatants revealed that CAFs secreted less PGE_2_ upon treatment with Cox-2 inhibitors (Figure 7C).

Based on these findings we hypothesized that reduced Cox-2 and PGE_2_ levels could lead to lower activity of the Akt and MapK pathways, both of which can be activated by PGE_2_ and mediate proliferation [43, 44]. Western Blot analyses of phosphorylated signal transduction intermediates revealed that phosphorylation of Erk was not influenced by PGE_2_ or Cox-2 inhibitors in CAFs (data not shown). In contrast, phosphorylation of Akt could be induced by PGE_2_ and was reduced upon Cox-2 inhibition (Figure 7D). Thus, the Cox-2 PGE_2_ axis could influence the proliferative state of CAFs via Akt. In order to test this hypothesis we incubated CAFs with PGE_2_, ASA and the well-described Akt inhibitor MK-2206 at a dose level showing almost complete inhibition of Akt phosphorylation (Figure 7D and Supplementary Figure S3A) [45]. These experiments showed that both the Akt inhibitor and ASA inhibited fibroblast proliferation when given alone and in combination with PGE_2_ (Figure 7E). However, when ASA and the Akt inhibitor were combined there was no additive effect of both treatments when incubated with or without PGE_2_ (Figure 7E). These findings indicate that the anti-proliferative effect of Cox-2 inhibition was mediated mainly by Akt signaling, because if Cox-2 inhibition would inhibit other pro-proliferative pathways one would expect an additive effect of Cox2 and Akt inhibition (Figure 7E).

The inhibitory effect of ASA on CAF proliferation was validated *in vivo* by performing morphometric analyses of BrdU^+^ CAFs in tumor sections treated with ASA. These analyses indicated reduced proliferation of CAFs upon treatment with ASA therapy (Figure 7F and G). Importantly, the inhibitory effect of ASA on CAF proliferation was preserved in the combination group and could at least partly explain the reduced numbers of CAFs upon treatment with ASA (Figure 4A and B).

### Cox-2 inhibition blocks migration of CAFs

**Figure 8:**
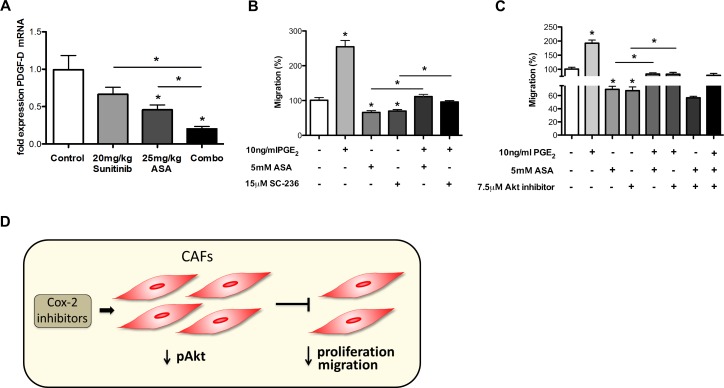
Cox-2 inhibition reduces migration of CAFs A, qRT-PCR analysis of PDGF-D mRNA expression in 4T1 tumor samples demonstrates a reduction upon combination of ASA and sunitinib treatments *in vivo* (Ct values normalized to GAPDH) (n = 7; *P < 0.01). B and C, migration assays performed with primary CAFs showing an induction of increased migration ability by PGE_2_ which is counteracted by ASA or SC-236 treatment (n = 3; *P < 0.005) (B). Akt inhibitor (MK-2206) and ASA inhibit the fibroblast migration alone or in combination with PGE_2_, but do not exert additive effects (n = 3; *P < 0.01) (C). D, Schematic outline of the mechanism that Cox-2 inhibitors can decrease Akt phosphorylation in CAFs, which inhibits their proliferation and migration. This effect might lead to smaller numbers of intratumoral CAFs.

In order to explore if Cox-2 inhibition influences known mediators involved in CAF recruitment into tumor tissues we quantified mRNA expression of TGFβ, interleukin 1β (IL1β), C-X-C motif chemokine 12 (CXCL12) also called SDF-1 (stromal cell-derived factor 1), and platelet derived growth factor D (PDGF-D) [38, 46, 47] in tumors treated with ASA and sunitinib. These experiments indicated that ASA reduced expression of TGFβ and PDGF-D both alone and in combination with sunitinib (Figure 5C and 8A) while expression levels of IL1β and CXCL12 mRNA were unchanged (data not shown).

Subsequently, we analyzed how Cox-2 and PGE_2_ influence migration of patient-derived CAFs by performing boyden chamber experiments. We found that migration of CAFs could be induced by PGE_2_ while it was inhibited by Cox-2 inhibitors (Figure 8B and Supplementary Figure S4). Importantly, ASA and SC-236 blocked PGE_2_-induced migration of CAFs to similar levels as observed in the control (Figure 8B).

Previous work documents that Akt signaling can promote migration of CAFs [48]. Thus, we were interested to determine whether the inhibitory effect of Cox-2 inhibitors on CAF migration was mediated via Akt. Therefore, in a similar experimental setup as described above we incubated CAFs with PGE_2_, Cox-2 inhibitors and MK-2206 both alone and in combination. Similar to our findings with regards to proliferation we found no additive reduction of CAF migration upon combining Cox-2 and Akt inhibition with and without PGE_2_ (Figure 8C). These data indicate that reduced CAF migration upon treatment with Cox-2 inhibitors is mainly mediated via Akt signaling.

Altogether, the reduction of intratumoral CAFs upon treatment with ASA can be explained by reduced recruitment/migration and by reduced proliferation (Figure 8D).

## DISCUSSION

This study yielded the following major findings (i) Cox-2 inhibitors and the anti-angiogenic drugs sunitinib and DC101 exert additive anti-cancer effects; (ii) these effects occur at standard but also at lower than typical therapeutic dose levels of anti-angiogenic drugs; (iii) anti-angiogenic drugs and Cox-2 inhibitors induce additive anti-angiogenic effects; (iv) ASA and high doses of sunitinib can block infiltration of tumors with activated CAFs; (v) treatment with ASA leads to decreased levels of intratumoral pro-angiogenic cytokines IL-6 and HGF which are not significantly modulated by sunitinib and (vi) Cox-2 inhibitors reduce proliferation and migration of CAFs via interfering with Akt signaling.

Our preclinical findings indicate that upregulation of Cox-2 and PGE_2_ occurs at standard dose levels of anti-angiogenic drugs. This upregulation is correlated with intra-tumoral hypoxia. The PGE_2_ upregulation appears to be more pronounced upon treatment with sunitinib compared to DC101 despite similar induction of Cox-2 mRNA upon both treatments. DC101 and sunitinib induce comparable level of intratumoral hypoxia. However, Cox-2 mRNA levels might not fully reflect the amount of the end product PGE_2_ because different mechanisms for regulation of Cox-2 enzymatic activity including protein degradation, allosteric regulation and protein phosphorylation were already described [49-51].

A large body of evidence in literature indicates that anti-angiogenic drugs at standard dose levels induce tumor hypoxia, which is considered a main driving force of anti-angiogenic therapy resistance [15, 52]. However, traditionally, drug doses used in mice and in humans are rather high, due to the long-lasting concept in oncology to treat patients with the maximum tolerated doses of drugs. This strategy might be counterproductive in the case of anti-angiogenic drugs (and possibly also other targeted agents). Clinical data also indicate that anti-angiogenic drugs induce hypoxia in tumor tissue of cancer patients [53]. Hypoxia can induce a plethora of resistance mechanisms including induction of pro-angiogenic cytokines besides the VEGF-axis or enhancement of cancer stem cell-like properties [54]. Our data indicate that combined inhibition of Cox-2 and anti-angiogenic treatments exert additive therapeutic effects. One might speculate that upregulation of Cox-2 and PGE_2_ might contribute to resistance against anti-angiogenic drugs which occur at standard (high) dose levels in an experimental breast cancer model. In line with this concept concomitant inhibition of Cox-2 increased efficacy of anti-angiogenic drugs. Prostaglandines and especially PGE_2_ can promote angiogenesis by different mechanisms besides the VEGF axis. PGE_2_ can bind directly to endothelial E-prostane surface receptors thereby enhancing endothelial cell migration, survival and proliferation [23]. In addition, Cox-2 is important for induction of FGF2-induced pro-angiogenic signals which are known to confer resistance to sunitinib [40]. Thus, the pro-angiogenic effect of Cox-2 is at least partly mediated by mechanisms which are distinct from the pro-angiogenic effect exerted via VEGF and its receptors. This alternative pro-angiogenic pathway would be inhibited by decreased Cox-2 expression and PGE_2_ levels and could hence be responsible for improved efficacy of anti-angiogenic treatments. In line with this concept we could detect an additive anti-angiogenic effect upon combined blockade of the VEGF pathway and Cox-2. A similar connection exists for other cytokines including placental growth factor and FGF family members that are upregulated upon hypoxia elicited by VEGF inhibitors [55, 56]. These mediators can support angiogenesis upon VEGF-blockade, thereby contributing to anti-angiogenic therapy resistance (Figure 8D).

Our data are in concordance with published literature indicating hypoxia-induced upregulation of Cox-2 in preclinical models of renal cancer upon treatment with high doses of sunitinib [57]. These data also indicated additive effects of sunitinib and the Cox-2 inhibitor celecoxib [57]. In this previous study tumor blood perfusion was not inhibited by celecoxib, thus the authors could not detect additive anti-angiogenic effects upon concomitant usage of celecoxib and sunitinib [57]. However, PGE_2_ levels were not measured and the underlying mechanism of enhanced efficacy was not investigated. It is also possible that the mechanism of action of Cox-2 inhibitors may vary in different cancer types.

Interestingly, in our study Cox-2 blockade and anti-angiogenic therapy also exerted additive anti-angiogenic effects at low doses of VEGF blockers at which PGE_2_ levels were not increased.

In order to analyze possible reasons for the additive anti-tumor effect at lower dose levels of anti-angiogenic drugs we carried out a profiling of the tumor microenvironment. We found that CAFs were reduced upon Cox-2 blockade and upon treatment with sunitinib. In addition, combinatorial treatments exerted additive effects on CAF reduction leading to an approximately 90% decrease in the number of tumor-infiltrating CAFs. ASA and higher dose levels of sunitinib (40 mg/kg and 60 mg/kg) could in addition reduce CAF-activation when applied as monotherapies. This inhibition of CAF recruitment and activation could be due to the inhibitory activity of sunitinib on PDGFR. A link between inhibition of PDGFR by imatinib was already demonstrated in CAFs isolated from colorectal metastases [59].

Anti-angiogenic agents can evoke side effects including deterioration of the patients quality of life [60]. Therefore, it would be of clinical interest to maintain/increase efficacy at lower dose levels. For instance, 40 mg/kg/d of sunitinib in a mouse already corresponds to the MTD of 75 mg/d of the drug in a patient [61, 62]. Of note, reductions of this dose level are often required due to intolerable side effects including nausea, asthenia and fatigue [63]. Our data indicate that adding 25 mg/kg ASA (corresponding to a daily dose of 150 mg in humans [64] to the treatment with a lower dose of sunitinib (20 mg/kg) exerts similar efficacy compared to monotherapy with 40 mg/kg sunitinib (Figure 1B, Supplementary Figure S5). Thus, combinatorial treatment with ASA might be useful to decrease the sunitinib dose upfront or to maintain efficacy in patients requiring dose reductions. Similarly, it was discussed that the combination of anti-angiogenic therapy and chemotherapy exert additive anti-angiogenic effects, which might allow dose reductions of both agents [58].

We found that ASA could reduce intratumoral levels of IL-6 and HGF while sunitinib also at higher dose levels was not significantly lowering expression levels of these cytokines. Both treatments reduced expression of TGFβ and FGF2 mRNA. Expression profiling of different cellular constituents of tumors indicated that HGF mRNA was predominantly expressed by CAFs and ECs while IL-6 mRNA was mainly expressed by tumor cells and by CAFs. FGF-2 and TGFβ were expressed by a wider range of cell types including TAMs and MDSCs. Hence, the observed reductions of TGFβ, FGF2, IL-6 and HGF can not only be due to the reduction of tumor-infiltrating CAFs but also due to quantitative and/or qualitative changes in other intratumoral cell populations such as reduced numbers of ECs due to an anti-angiogenic effect of the treatments.

IL-6 and HGF were both shown to be capable to induce angiogenesis in the presence of VEGF inhibitors and could thus lower the efficacy of anti-angiogenic agents. This might explain the enhanced treatment efficacy upon combination of ASA and anti-angiogenic drugs [65]. Another reason could be that both treatments are additive because they inhibit different cancer-promoting pathways. Further work is necessary in order to determine the underlying mechanisms as both Cox-2 inhibitors and VEGF(R)Is exert pleiotropic effects on tumor cells and their microenvironment [66]. Especially sunitinib targets many different kinases thus our findings could also be attributed to other mechanisms besides the drug's effects on CAFs and ECs. In addition, our results could be influenced by the triple negative phenotype of the tumor cells. Further work is necessary to elucidate whether the observed findings also hold true in hormone receptor and/or HER2-positive cell lines.

In our tumor models VEGF expression was not significantly reduced by Cox-2 inhibitors. Therefore, the observed additive effect of concomitant treatment with VEGF- and Cox-2 inhibitors is most likely not due to a reduction of VEGF expression induced by the latter. However, published literature indicates downregulation of VEGF mRNA expression in some tumor models after blockade of Cox-2. Hence, the effect of Cox-2 on VEGF expression might vary in a context and cell-type specific manner and further work is necessary in order to determine the underlying mechanisms. Even though VEGF-A could be downregulated upon Cox-2 inhibition, its signaling would be inhibited by the pan-VEGFR inhibitor sunitinib [67]. Thus, it appears rather unlikely that a downregulation of VEGF-A upon Cox-2 inhibition can be responsible for the additive effects of sunitinib and Cox-2 blockade. Concerning DC101 it is possible that due to decreased VEGF-A levels the pro-angiogenic signaling via VEGFR-1 could be reduced. However, the pro-angiogenic signal transmitted by VEGFR-1 is about 7-fold weaker compared to VEGFR-2 [68] and thus it might not be sufficient to explain the phenotype.

The observed reduction of CAFs could be explained by their decreased proliferation and/or migration. *In vitro* assays indicated that PGE_2_ increased CAF proliferation and migration, which can be inhibited by Cox-2 blockers. Importantly, these drugs can also decrease CAF proliferation and migration in baseline conditions indicating that this process does not require PGE_2_ induction. Upon analysis of candidate molecular mechanisms involved in CAF proliferation and migration we found that phosphorylation of Akt but not Erk was inhibited by Cox-2 blockade. In addition, Cox-2 inhibition and Akt blockade did not exert additive effects on proliferation or migration indicating that the effect of Cox-2 inhibition on proliferation is mainly mediated by interfering with Akt signaling.

In concordance with published literature the decreased Akt phosphorylation could at least partially explain the reduced CAF proliferation and migration [48]. The finding that Cox-2 inhibitors reduce CAF proliferation by interfering with Akt signaling is novel. Available literature indicates that Cox-2 inhibitors induce rather than inhibit the proliferation of other types of fibroblasts including lung fibroblasts [69]. Hence, the effect of Cox-2 inhibition on fibroblasts seems to be context-dependent. Further work is necessary in order to determine the effect of Cox-2 inhibitors on CAFs in other tumor types besides breast cancer. In addition, we found that TGFβ and platelet derived growth factor B (PDGF-B), both of which are capable to induce recruitment of CAFs were reduced upon treatment with Cox-2 inhibitors.

In summary, concomitant anti-angiogenic therapy and Cox-2 blockade represents a novel clinically applicable approach to increase efficacy of anti-angiogenic drugs at high and low dose levels. These findings may also have significant implications for further clinical trials in breast cancer and possibly in other cancer types where anti-angiogenic approaches are used.

## MATERIAL AND METHODS

### Animals

8 weeks old female Balb/C mice were purchased from Charles River Laboratories International (Sulzfeld, Germany). All animal experiments were carried out according to the institutional guidelines for the welfare of animals in experimental neoplasia and were approved by the local licensing authority (Behörde für Soziales, Familie, Gesundheit, Verbraucherschutz; Amt für Gesundheit und Verbraucherschutz, Hamburg, Germany, project number 98/10). Housing, breeding and experiments were performed under a 12h light – 12h dark cycle and standard laboratory conditions (22 ± 1°C, 55% humidity, food and water ad libitum and 150–400 lx light intensity during the light phase).

### Reagents

ASA or aspirin (acetylsalicylic acid) was purchased from Sigma and diluted in 0.9% NaCl solution with dimethyl sulfoxide (DMSO). The final concentration of DMSO in the medium was 1/1000 (v/v). The solution was freshly prepared. SC-236 was purchased from Cayman Chemical (Ann Arbor, MI, USA) and was dissolved in ethanol with phosphate buffered saline pH 7.6 (PBS) in a 1:4 solution of ethanol:PBS, which was stored at −20°C. Sunitinib Malate was obtained from Pfizer (Pfizer Inc., New York, NY, USA) and was suspended in carboxymethylcellulose (CMC) solution (0.5% CMC, 1.8% NaCl, 0.4% Tween 80, and 0.9% benzyl alcohol in distilled water) for *in vivo* experiments. Drug aliquots were prepared once weekly and kept in the dark at 4°C. DC101 was obtained from ImClone Systems (Branchburg, NJ, USA) and was diluted in PBS. Aliquots were stored at −20°C. PGE_2_ was bought from Cayman Chemical, dissolved in DMSO and stored at −20°C. The Akt inhibitor MK-2206 was purchased from Merck Millipore (Darmstadt, Germany), dissolved in PBS with 20% ethanol and stored at −20°C.

### Cells and culture conditions

The mouse breast cancer cell line 4T1 was obtained from Peter Carmeliet (VIB Vesalius Research Centre, K.U. Leuven, Belgium) and murine 66cl4 cells were provided by Dr. Fred Miller (Karmanos Cancer Institute and Wayne State University, Detroit, MI). They were cultured in RPMI-1640 and DMEM medium, respectively. The cell lines were not authenticated by the authors as cell authentication testing can be conducted only on human cell lines. Primary CAFs were isolated from tumor tissue of n=2 lung cancer patients as described [70] (kindly provided by Iñigo Martinez-Zubiaurre, Arctic University of Norway, Tromsø, Norway) and cultured in DMEM. The embryonic fibroblast cell line MRC-5 was cultured in MEM supplemented with 1% sodium pyruvate. All cells were cultured in medium supplemented with 10% FCS, 1% Penicillin/Streptomycin (P/S) and 1% L-glutamine (all cell culture reagents were purchased from Invitrogen, Darmstadt, Germany).

### Treatments

Primary CAFs and MRC-5 cells were starved in serum-free medium over night followed by treatment with different reagents. For qRT-PCR and WST-1 assays 1 × 10^5^ or 9 × 10^3^ cells were seeded in 6 well-plates or 96 well-plates, respectively, and incubated for 48 hours in serum free-medium containing either or a combination of 10 ng/ml PGE_2_, 5 mM ASA, 15 μM SC-236 and 7.5 μM Akt inhibitor. For migration assays 1.1 × 10^4^ cells were seeded in the migration inserts of 24 well-plates and treated with 10 ng/ml PGE_2_, 5 mM ASA, 15 μM SC-236 and 7.5 μM Akt inhibitor for 24 hours in serum free medium. For western blotting 3 × 10^6^ cells were seeded and incubated with 10 ng/ml PGE_2_, 5 mM ASA, 15 μM SC-236, 5 and 7.5 μM Akt inhibitor for 3 hours.

### Cell growth

Cell viability was assessed by WST-1 assay (Roche Applied Science, Mannheim, Germany) or by Trypan Blue exclusion was carried out as previously described [71].

### Breast cancer model and treatments

5×10^5^ 4T1 or 1×10^6^ 66cl4 murine breast cancer cells (both hormone receptor- and HER2-negative [72, 73]) were orthotopically implanted into the second mammary gland of syngeneic Balb/C mice. The animals were randomized according to tumor size after 7-10 days (mean tumor size 80-150 mm^3^) and were treated with daily intraperitoneal (i.p.) injections of 25 and 100 mg/kg ASA. Treatment was given three times per week with 1.5 mg/kg SC-236 and 10, 15 or 40 mg/kg DC101 i.p. Sunitinib 10, 20, 40 or 60 mg/kg was administered by oral gavage once per day. Combinatory treatments have been carried out as described in the graphs and all treatments were started on the same day on which both drugs were administered. Tumor growth was monitored by calliper. Tumor volume was calculated according to the formula V=(longer length^2^ x shorter length)/2. Mice were sacrificed according to ethical regulations when the first tumor in the control group reached the maximum allowed size of 1500 mm^3^. After sacrification of mice, tumors were weighed, pieces were embedded in paraffin for further immunohistochemistry (IHC) analysis and fresh tissue was frozen for protein and RNA extractions.

### FACS sorting

4T1 cells were stably transduced with the lentiviral vector LeGO-G2 expressing enhanced *green fluorescent protein* (eGFP) [74] and cells were injected into the mice as described above. Tumors were resected, cut into small pieces and digested in 10 ml digestion buffer (collagenase A and DNase (from Roche)) for 1 hour at 37°C. A single cell suspension was obtained by mincing the digested tumor through a 70 μm-pore cell strainer. The cells were incubated with Fc-block (anti-CD16/32) from BioLegend (San Diego, CA, USA) and stained with the following antibodies: anti-F4/80-APC, anti-CD11b-PE-Cy7, anti-Ly6C-PE, anti-Ly6G-PercP 5.5, CD45-APC, CD31-PE-Cy7, all purchased from eBioscience (Frankfurt am Main, Germany), and anti-PDGFR-PE from BioLegend. Specificity of antibodies was determined by using appropriate isotype controls. Dead cells were excluded from analysis using 7-AAD or DAPI. Multicolor-flow cytometry was used to sort tumor cells (GFP^+^/CD45^−^), tumor-associated macrophages (TAMs) (F4/80^+^/CD11b^+^), endothelial cells (ECs) (CD31^+^/CD45^−^/GFP^−^), cancer-associated fibroblast (CAFs) (PDGFR^+^/CD45^−^/GFP^−^) granulocytic myeloid-derived suppressor cells (gMDSCs) (CD11b^+^/Ly6G^+^Ly6C^low/−^) and monocytic MDSCs (mMDSCs) (CD11b^+^/Ly6C^+^Ly6G^−^). FACS sorting was performed on FACS Aria IIIu using the FACS Diva software version 6.1.3. (both from BD Biosciences (Bedford, MA, USA)). Sorted cells were recollected in RNA lysis buffer (Ambion, Life Techonologies, Darmstadt, Germany).

### ELISA

PGE_2_ levels were determined in tumor lysates by a commercially available competitive ELISA for PGE_2_ (R&D Systems, Minneapolis, MN, USA). Tumors were mechanically disaggregated in 0.1 M potassium phosphate buffer containing protease inhibitors prior to homogenization by sonication. The protein concentration of tumor lysates was determined by the Bradford protein assay and a total protein amount of 3 μg was used. PGE_2_ levels in cell culture supernatants were determined using a competitive ELISA for PGE_2_ from Enzo Life Science (Famingdale, NY, USA). ELISA assays were performed as described by the manufacturers instructions. The absorbance was measured by a microplate reader (Tecan). The PGE_2_ concentrations were calculated using a four parameter logistic (4-PL) curve. Each tumor, supernatant sample and standard was analyzed in triplicate measurements.

### RT-PCR

Total cellular mRNA was extracted from sorted or cultured cells using the Ambion PureLink® RNA Mini Kit followed by cDNA synthesis using first strand cDNA synthesis kit (Thermo Fisher Scientific, distributed through Life Technologies) according to manufacturer's instructions. The relative levels of gene expression for each experimental sample were performed by quantitative real-time RT-PCR (qRT-PCR). mRNA levels of target genes were quantified using the SYBR Green qPCR Master Mix and ABI 7500 instrument (Applied Biosystems, distributed through Life Technologies). Expression of GAPDH served as an endogenous control. All Primers are shown in Supplemental Table 1 and were synthesized by Eurofins MWG, Ebersberg, Germany. The relative mRNA levels in each sample were calculated based on their threshold cycle (Ct) normalized by their respective Ct value of GAPDH using the ΔΔCt-method.

### Histology and morphometric analysis

Tumors were resected, fixed overnight in 1% parafolmaldehyde at 4°C and embedded in paraffin. Paraffin-embedded tissues were sectioned at 4μm, deparaffinized in xylene and rehydrated through a series of graded ethanol to distilled water. Antigen retrieval was performed in citrate buffer (Dako, distributed through Agilent Technologies, Glostrup, Denmark) using a steamer. After cooling to room temperature, sections were incubated with 0.3% hydrogen peroxide to inactivate endogenous peroxidases followed by washes with TBS buffer. Incubation with 1% bovine serum albumin (BSA) and 0.3% tritonX-100 was performed to inhibit unspecific binding, followed by incubation with antibodies for CD31 (1:200, Abcam, Cambridge, United Kingdom) or Cox-2 (1:500, Abcam) overnight at 4°C. Sections were then incubated with horseradish peroxidase conjugated secondary antibodies, followed by incubation with DAB solution, and counterstained with hematoxylin. Immunofluorescence staining of CAFs was performed in paraffin-embedded tissues after deparaffinization, antigen retrieval and inhibition of unspecific binding sites as described above. Incubation with 1% BSA in TNT buffer was performed to permeabilize sections prior to overnight incubation with antibodies for vimentin (1:500, Abcam) and *alpha smooth muscle actin* (α-SMA) conjugated with Cy3 (1:200, Sigma-Aldrich) or vimentin (1:1000, Novus) and BrdU (bromodeoxyuridine) (1:200, AbD Serotec, Kidlington, United Kingdom) at 4°C. Sections were then washed with TNT buffer and incubated with secondary antibodies conjugated with Alexa Fluor dyes (1:200, Dianova, Hamburg, Germany and Molecular Probes distributed through Life Technologies Darmstadt, Germany) as well as with DAPI to visualize nuclei. The labeled sections were mounted with glass coverslips on slides and imaged using a Zeiss Axio Scope.A1 (Carl Zeiss Microscopy, Jena, Germany) for IHC or a Leica DM5000 B (Leica Microsystems, Wetzlar, Germany) for immunofluorescence (IF). Image analysis was performed using the imaging software AxioVision (Carl Zeiss Microscopy). Manual counting of immunostained cells in the tumor stroma was performed in 10-12 fields of each tumor section (20x magnification). Microvessel and CAF densities were calculated as the number of counted CD31^+^ and as α-SMA^+^ and/or Vimentin^+^ cells per total area of analyzed tumor tissue, respectively. Hypoxia was detected in paraffin sections of tumor tissue by using the pimonidazole (PIMO) method as described [55]. Pictures were taken with 10x magnification using an AxioScope.A1 microscope and Axiovision software. The PIMO^+^ area was manually assessed after IHC staining and digital image acquisition. Subsequently, the fraction of PIMO^+^ area was calculated relative to the total tumor area. Areas of necrosis were excluded from all analyses.

### Migration assay

The kit *BD BioCoat Matrigel invasion chambers* (BD Biosciences) was used. To assess, cellular migration inserts without matrigel (8 μm pore size) were utilized. 1.1 × 10^4^ cells were seeded onto the inserts containing 400 μl of serum-free medium. The insert was placed in a 24-well plate and 400 μl of serum free medium were added to the bottom of each well. The cells were kept in culture for 24 hours in standard conditions with different treatments. After this time, the cells that had not migrated were gently removed by washing with a cotton swab. The cells that remained in the inserts were fixed with 100% methanol for 2 minutes and stained with cristal violet. The stained cells were imaged using an an Axio-Scope.A1 microscope (Carl Zeiss Microscopy) and counted to evaluate their migratory activity.

### Western blot analysis

To assess levels of protein expression cells were lysed in RIPA buffer with protease and phosphatase inhibitors. Western Blotting was carried out as previously described [71]. The pAkt and tAkt antibodies were purchased from Cell Signaling (distributed through New England Biolabs GmbH, Frankfurt am Main, Germany). β-Actin antibody was purchased from Santa Cruz Biotechnology (Heidelberg, Germany). Incubation of membranes after SDS-Gel electrophoresis and transfer on nitrocellulose membranes with the primary antibodies (1:200 to 1:1000) was performed overnight at 4°C. After washing, the membranes were incubated with the corresponding peroxidase-conjugated secondary antibodies for 1 hour at room temperature. Blots were developed using the ECL Western Blotting analysis system (GE Healthcare, Freiburg, Germany) and visualized by developing of standard X-ray films.

### Statistics

Experiments were performed at least in triplicates. Data represent mean ± SEM (standard error of the means) of representative experiments, unless otherwise stated. Statistical significance was calculated by Student's T-Test unless otherwise stated. To study dependence of numerical dependent parameters of n>2 categorial variables ANOVA was used where indicated. All statistical analyses have been performed with GraphPad Prism 5.0 (GraphPad Software, La Jolla, CA, USA).

## SUPPLEMENTARY MATERIAL, FIGURES AND TABLE



## References

[1] Hanahan D, Weinberg RA (2011). Hallmarks of cancer: the next generation. Cell.

[2] Folkman J (1971). Tumor angiogenesis: therapeutic implications. N Engl J Med.

[3] Carmeliet P (2005). Angiogenesis in life, disease and medicine. Nature.

[4] Ferrara N, Kerbel RS (2005). Angiogenesis as a therapeutic target. Nature.

[5] Kerbel RS (2008). Tumor angiogenesis. N Engl J Med.

[6] Welti J, Loges S, Dimmeler S, Carmeliet P (2013). Recent molecular discoveries in angiogenesis and antiangiogenic therapies in cancer. The Journal of clinical investigation.

[7] Reinacher-Schick A, Pohl M, Schmiegel W (2008). Drug insight: antiangiogenic therapies for gastrointestinal cancers--focus on monoclonal antibodies. Nat Clin Pract Gastroenterol Hepatol.

[8] Grothey A, Galanis E (2009). Targeting angiogenesis: progress with anti-VEGF treatment with large molecules. Nature reviews Clinical oncology.

[9] Meadows KL, Hurwitz HI (2012). Anti-VEGF therapies in the clinic. Cold Spring Harbor perspectives in medicine.

[10] Kerbel RS (2011). Reappraising antiangiogenic therapy for breast cancer. Breast.

[11] Wilke H. VCE, Oh S.C., Bodoky G., Shimada Y., Hironaka S., Sugimoto N., Lipatov O.N, Kim T.Y, Cunningham D, Ohtsu A, Rougier P, Emig M, Carlesi R, Chandrawansa K, Muro K (2014). (2014). RAINBOW: A global, phase III, randomized, double-blind study of ramucirumab plus paclitaxel versus placebo plus paclitaxel in the treatment of metastatic gastroesophageal junction (GEJ) and gastric adenocarcinoma following disease progression on first-line platinum- and fluoropyrimidine-containing combination therapy rainbow IMCL CP12-0922 (I4T-IE-JVBE). Gastrointestinal Cancers Symposium.

[12] Cortazar P, Justice R, Johnson J, Sridhara R, Keegan P, Pazdur R (2012). US Food and Drug Administration approval overview in metastatic breast cancer. Journal of clinical oncology : official journal of the American Society of Clinical Oncology.

[13] Kollmannsberger C, Soulieres D, Wong R, Scalera A, Gaspo R, Bjarnason G (2007). Sunitinib therapy for metastatic renal cell carcinoma: recommendations for management of side effects. Canadian Urological Association journal = Journal de l'Association des urologues du Canada.

[14] Bergers G, Hanahan D (2008). Modes of resistance to anti-angiogenic therapy. Nature reviews Cancer.

[15] Loges S, Schmidt T, Carmeliet P (2010). Mechanisms of resistance to anti-angiogenic therapy and development of third-generation anti-angiogenic drug candidates. Genes Cancer.

[16] Shojaei F, Wu X, Malik AK, Zhong C, Baldwin ME, Schanz S, Fuh G, Gerber HP, Ferrara N (2007). Tumor refractoriness to anti-VEGF treatment is mediated by CD11b+Gr1+ myeloid cells. Nature biotechnology.

[17] Murdoch C, Muthana M, Coffelt SB, Lewis CE (2008). The role of myeloid cells in the promotion of tumour angiogenesis. Nature reviews Cancer.

[18] Paez-Ribes M, Allen E, Hudock J, Takeda T, Okuyama H, Vinals F, Inoue M, Bergers G, Hanahan D, Casanovas O (2009). Antiangiogenic therapy elicits malignant progression of tumors to increased local invasion and distant metastasis. Cancer cell.

[19] Maione F, Capano S, Regano D, Zentilin L, Giacca M, Casanovas O, Bussolino F, Serini G, Giraudo E (2012). Semaphorin 3A overcomes cancer hypoxia and metastatic dissemination induced by antiangiogenic treatment in mice. The Journal of clinical investigation.

[20] Gaustad JV, Simonsen TG, Leinaas MN, Rofstad EK (2012). Sunitinib treatment does not improve blood supply but induces hypoxia in human melanoma xenografts. BMC cancer.

[21] Schmedtje JF, Ji YS, Liu WL, DuBois RN, Runge MS (1997). Hypoxia induces cyclooxygenase-2 via the NF-kappaB p65 transcription factor in human vascular endothelial cells. The Journal of biological chemistry.

[22] Lee JJ, Natsuizaka M, Ohashi S, Wong GS, Takaoka M, Michaylira CZ, Budo D, Tobias JW, Kanai M, Shirakawa Y, Naomoto Y, Klein-Szanto AJ, Haase VH, Nakagawa H (2010). Hypoxia activates the cyclooxygenase-2-prostaglandin E synthase axis. Carcinogenesis.

[23] Ruegg C, Dormond O, Mariotti A (2004). Endothelial cell integrins and COX-2: mediators and therapeutic targets of tumor angiogenesis. Biochim Biophys Acta.

[24] Howe LR, Subbaramaiah K, Patel J, Masferrer JL, Deora A, Hudis C, Thaler HT, Muller WJ, Du B, Brown AM, Dannenberg AJ (2002). Celecoxib, a selective cyclooxygenase 2 inhibitor, protects against human epidermal growth factor receptor 2 (HER-2)/neu-induced breast cancer. Cancer research.

[25] Yoshinaka R, Shibata MA, Morimoto J, Tanigawa N, Otsuki Y (2006). COX-2 inhibitor celecoxib suppresses tumor growth and lung metastasis of a murine mammary cancer. Anticancer research.

[26] Connolly EM, Harmey JH, O'Grady T, Foley D, Roche-Nagle G, Kay E, Bouchier-Hayes DJ (2002). Cyclo-oxygenase inhibition reduces tumour growth and metastasis in an orthotopic model of breast cancer. British journal of cancer.

[27] Welti JC, Powles T, Foo S, Gourlaouen M, Preece N, Foster J, Frentzas S, Bird D, Sharpe K, van Weverwijk A, Robertson D, Soffe J, Erler JT, Pili R, Springer CJ, Mather SJ (2012). Contrasting effects of sunitinib within *in vivo* models of metastasis. Angiogenesis.

[28] Prewett M, Huber J, Li Y, Santiago A, O'Connor W, King K, Overholser J, Hooper A, Pytowski B, Witte L, Bohlen P, Hicklin DJ (1999). Antivascular endothelial growth factor receptor (fetal liver kinase 1) monoclonal antibody inhibits tumor angiogenesis and growth of several mouse and human tumors. Cancer research.

[29] Kaidi A, Qualtrough D, Williams AC, Paraskeva C (2006). Direct transcriptional up-regulation of cyclooxygenase-2 by hypoxia-inducible factor (HIF)-1 promotes colorectal tumor cell survival and enhances HIF-1 transcriptional activity during hypoxia. Cancer research.

[30] Yao R, Rioux N, Castonguay A, You M (2000). Inhibition of COX-2 and induction of apoptosis: two determinants of nonsteroidal anti-inflammatory drugs' chemopreventive efficacies in mouse lung tumorigenesis. Experimental lung research.

[31] Crawford Y, Kasman I, Yu L, Zhong C, Wu X, Modrusan Z, Kaminker J, Ferrara N (2009). PDGF-C mediates the angiogenic and tumorigenic properties of fibroblasts associated with tumors refractory to anti-VEGF treatment. Cancer cell.

[32] Orimo A, Gupta PB, Sgroi DC, Arenzana-Seisdedos F, Delaunay T, Naeem R, Carey VJ, Richardson AL, Weinberg RA (2005). Stromal fibroblasts present in invasive human breast carcinomas promote tumor growth and angiogenesis through elevated SDF-1/CXCL12 secretion. Cell.

[33] Li F, Fan C, Zeng B, Zhang C, Chai Y, Liu S, Ouyang Y (2012). Celecoxib suppresses fibroblast proliferation and collagen expression by inhibiting ERK1/2 and SMAD2/3 phosphorylation. Molecular medicine reports.

[34] Quante M, Tu SP, Tomita H, Gonda T, Wang SS, Takashi S, Baik GH, Shibata W, Diprete B, Betz KS, Friedman R, Varro A, Tycko B, Wang TC (2011). Bone marrow-derived myofibroblasts contribute to the mesenchymal stem cell niche and promote tumor growth. Cancer cell.

[35] Polanska UM, Orimo A (2013). Carcinoma-associated fibroblasts: non-neoplastic tumour-promoting mesenchymal cells. Journal of cellular physiology.

[36] Kalluri R, Zeisberg M (2006). Fibroblasts in cancer. Nature reviews Cancer.

[37] Shimoda M, Mellody KT, Orimo A (2010). Carcinoma-associated fibroblasts are a rate-limiting determinant for tumour progression. Seminars in cell & developmental biology.

[38] Kojima Y, Acar A, Eaton EN, Mellody KT, Scheel C, Ben-Porath I, Onder TT, Wang ZC, Richardson AL, Weinberg RA, Orimo A (2010). Autocrine TGF-beta and stromal cell-derived factor-1 (SDF-1) signaling drives the evolution of tumor-promoting mammary stromal myofibroblasts. Proceedings of the National Academy of Sciences of the United States of America.

[39] Karaman MW, Herrgard S, Treiber DK, Gallant P, Atteridge CE, Campbell BT, Chan KW, Ciceri P, Davis MI, Edeen PT, Faraoni R, Floyd M, Hunt JP, Lockhart DJ, Milanov ZV, Morrison MJ (2008). A quantitative analysis of kinase inhibitor selectivity. Nature biotechnology.

[40] Welti JC, Gourlaouen M, Powles T, Kudahetti SC, Wilson P, Berney DM, Reynolds AR (2011). Fibroblast growth factor 2 regulates endothelial cell sensitivity to sunitinib. Oncogene.

[41] Wei D, Wang L, He Y, Xiong HQ, Abbruzzese JL, Xie K (2004). Celecoxib inhibits vascular endothelial growth factor expression in and reduces angiogenesis and metastasis of human pancreatic cancer via suppression of Sp1 transcription factor activity. Cancer research.

[42] Tortora G, Caputo R, Damiano V, Melisi D, Bianco R, Fontanini G, Veneziani BM, De Placido S, Bianco AR, Ciardiello F (2003). Combination of a selective cyclooxygenase-2 inhibitor with epidermal growth factor receptor tyrosine kinase inhibitor ZD1839 and protein kinase A antisense causes cooperative antitumor and antiangiogenic effect. Clinical cancer research : an official journal of the American Association for Cancer Research.

[43] Sobolewski C, Cerella C, Dicato M, Ghibelli L, Diederich M (2010). The role of cyclooxygenase-2 in cell proliferation and cell death in human malignancies. International journal of cell biology.

[44] Vo BT, Morton D, Komaragiri S, Millena AC, Leath C, Khan SA (2013). TGF-beta effects on prostate cancer cell migration and invasion are mediated by PGE2 through activation of PI3K/AKT/mTOR pathway. Endocrinology.

[45] Hirai H, Sootome H, Nakatsuru Y, Miyama K, Taguchi S, Tsujioka K, Ueno Y, Hatch H, Majumder PK, Pan BS, Kotani H (2010). MK-2206, an allosteric Akt inhibitor, enhances antitumor efficacy by standard chemotherapeutic agents or molecular targeted drugs *in vitro* and *in vivo*. Molecular cancer therapeutics.

[46] De Wever O, Westbroek W, Verloes A, Bloemen N, Bracke M, Gespach C, Bruyneel E, Mareel M (2004). Critical role of N-cadherin in myofibroblast invasion and migration *in vitro* stimulated by colon-cancer-cell-derived TGF-beta or wounding. Journal of cell science.

[47] Donovan J, Shiwen X, Norman J, Abraham D (2013). Platelet-derived growth factor alpha and beta receptors have overlapping functional activities towards fibroblasts. Fibrogenesis & tissue repair.

[48] Al-Ansari MM, Hendrayani SF, Tulbah A, Al-Tweigeri T, Shehata AI, Aboussekhra A (2012). p16INK4A represses breast stromal fibroblasts migration/invasion and their VEGF-A-dependent promotion of angiogenesis through Akt inhibition. Neoplasia.

[49] Dong L, Vecchio AJ, Sharma NP, Jurban BJ, Malkowski MG, Smith WL (2011). Human cyclooxygenase-2 is a sequence homodimer that functions as a conformational heterodimer. The Journal of biological chemistry.

[50] Garavito RM, Malkowski MG, DeWitt DL (2002). The structures of prostaglandin endoperoxide H synthases-1 and -2. Prostaglandins & other lipid mediators.

[51] Alexanian A, Miller B, Chesnik M, Mirza S, Sorokin A (2014). Post-translational regulation of COX2 activity by FYN in prostate cancer cells. Oncotarget.

[52] Loges S, Mazzone M, Hohensinner P, Carmeliet P (2009). Silencing or fueling metastasis with VEGF inhibitors: antiangiogenesis revisited. Cancer cell.

[53] Garcia-Foncillas J, Martinez P, Lahuerta A, Llombart Cussac A, Garcia Gonzalez M, Sanchez Gomez RM, Alvarez I, Anton A, Illarramendi JJ, De Juan A, Galve Calvo E, Plazaola A, Morales S, Hernando B, Lao J, Boni V (2012). Dynamic contrast-enhanced MRI versus 18F-misonidazol-PET/CT to predict pathologic response in bevacizumab-based neoadjuvant therapy in breast cancer. Journal of clinical oncology : official journal of the American Society of Clinical Oncology.

[54] Conley SJ, Gheordunescu E, Kakarala P, Newman B, Korkaya H, Heath AN, Clouthier SG, Wicha MS (2012). Antiangiogenic agents increase breast cancer stem cells via the generation of tumor hypoxia. Proceedings of the National Academy of Sciences of the United States of America.

[55] Fischer C, Jonckx B, Mazzone M, Zacchigna S, Loges S, Pattarini L, Chorianopoulos E, Liesenborghs L, Koch M, De Mol M, Autiero M, Wyns S, Plaisance S, Moons L, van Rooijen N, Giacca M (2007). Anti-PlGF inhibits growth of VEGF(R)-inhibitor-resistant tumors without affecting healthy vessels. Cell.

[56] Casanovas O, Hicklin DJ, Bergers G, Hanahan D (2005). Drug resistance by evasion of antiangiogenic targeting of VEGF signaling in late-stage pancreatic islet tumors. Cancer cell.

[57] Wang X, Zhang L, O'Neill A, Bahamon B, Alsop DC, Mier JW, Goldberg SN, Signoretti S, Atkins MB, Wood CG, Bhatt RS (2013). Cox-2 inhibition enhances the activity of sunitinib in human renal cell carcinoma xenografts. British journal of cancer.

[58] Blagosklonny MV (2005). How Avastin potentiates chemotherapeutic drugs: action and reaction in antiangiogenic therapy. Cancer biology & therapy.

[59] Mueller L, Goumas FA, Himpel S, Brilloff S, Rogiers X, Broering DC (2007). Imatinib mesylate inhibits proliferation and modulates cytokine expression of human cancer-associated stromal fibroblasts from colorectal metastases. Cancer letters.

[60] Chen HX, Cleck JN (2009). Adverse effects of anticancer agents that target the VEGF pathway. Nature reviews Clinical oncology.

[61] Faivre S, Delbaldo C, Vera K, Robert C, Lozahic S, Lassau N, Bello C, Deprimo S, Brega N, Massimini G, Armand JP, Scigalla P, Raymond E (2006). Safety, pharmacokinetic, and antitumor activity of SU11248, a novel oral multitarget tyrosine kinase inhibitor, in patients with cancer. Journal of clinical oncology : official journal of the American Society of Clinical Oncology.

[62] Mendel DB, Laird AD, Xin X, Louie SG, Christensen JG, Li G, Schreck RE, Abrams TJ, Ngai TJ, Lee LB, Murray LJ, Carver J, Chan E, Moss KG, Haznedar JO, Sukbuntherng J (2003). *In vivo* antitumor activity of SU11248, a novel tyrosine kinase inhibitor targeting vascular endothelial growth factor and platelet-derived growth factor receptors: determination of a pharmacokinetic/pharmacodynamic relationship. Clinical cancer research : an official journal of the American Association for Cancer Research.

[63] Kollmannsberger C, Bjarnason G, Burnett P, Creel P, Davis M, Dawson N, Feldman D, George S, Hershman J, Lechner T, Potter A, Raymond E, Treister N, Wood L, Wu S, Bukowski R (2011). Sunitinib in metastatic renal cell carcinoma: recommendations for management of noncardiovascular toxicities. The oncologist.

[64] Jeong KH, Kim JY, Choi YS, Lee MY, Kim SY (2013). Influence of aspirin on pilocarpine-induced epilepsy in mice. The Korean journal of physiology & pharmacology : official journal of the Korean Physiological Society and the Korean Society of Pharmacology.

[65] Shojaei F, Lee JH, Simmons BH, Wong A, Esparza CO, Plumlee PA, Feng J, Stewart AE, Hu-Lowe DD, Christensen JG (2010). HGF/c-Met acts as an alternative angiogenic pathway in sunitinib-resistant tumors. Cancer research.

[66] Grosch S, Maier TJ, Schiffmann S, Geisslinger G (2006). Cyclooxygenase-2 (COX-2)-independent anticarcinogenic effects of selective COX-2 inhibitors. Journal of the National Cancer Institute.

[67] Kumar R, Crouthamel MC, Rominger DH, Gontarek RR, Tummino PJ, Levin RA, King AG (2009). Myelosuppression and kinase selectivity of multikinase angiogenesis inhibitors. British journal of cancer.

[68] Kroll J, Waltenberger J (1997). The vascular endothelial growth factor receptor KDR activates multiple signal transduction pathways in porcine aortic endothelial cells. The Journal of biological chemistry.

[69] Huang S, Wettlaufer SH, Hogaboam C, Aronoff DM, Peters-Golden M (2007). Prostaglandin E(2) inhibits collagen expression and proliferation in patient-derived normal lung fibroblasts via E prostanoid 2 receptor and cAMP signaling. American journal of physiology Lung cellular and molecular physiology.

[70] Hellevik T, Pettersen I, Berg V, Winberg JO, Moe BT, Bartnes K, Paulssen RH, Busund LT, Bremnes R, Chalmers A, Martinez-Zubiaurre I (2012). Cancer-associated fibroblasts from human NSCLC survive ablative doses of radiation but their invasive capacity is reduced. Radiation oncology.

[71] Ben-Batalla I, Schultze A, Wroblewski M, Erdmann R, Heuser M, Waizenegger JS, Riecken K, Binder M, Schewe D, Sawall S, Witzke V, Cubas-Cordova M, Janning M, Wellbrock J, Fehse B, Hagel C (2013). Axl, a prognostic and therapeutic target in acute myeloid leukemia mediates paracrine crosstalk of leukemia cells with bone marrow stroma. Blood.

[72] Kaur P, Nagaraja GM, Zheng H, Gizachew D, Galukande M, Krishnan S, Asea A (2012). A mouse model for triple-negative breast cancer tumor-initiating cells (TNBC-TICs) exhibits similar aggressive phenotype to the human disease. BMC cancer.

[73] Phoenix KN, Vumbaca F, Fox MM, Evans R, Claffey KP (2010). Dietary energy availability affects primary and metastatic breast cancer and metformin efficacy. Breast cancer research and treatment.

[74] Weber K, Mock U, Petrowitz B, Bartsch U, Fehse B (2010). Lentiviral gene ontology (LeGO) vectors equipped with novel drug-selectable fluorescent proteins: new building blocks for cell marking and multi-gene analysis. Gene therapy.

