# Supplementary material for: Cyclooxygenase-2 blockade can improve efficacy of VEGF-targeting drugs
Source: Oncotarget. 2015 Jan 31;6(8):6341–58. doi: 10.18632/oncotarget.3437 (PMC4467441; doi:10.18632/oncotarget.3437)
Supplement: Supplementary file 1 [file oncotarget-06-6341-s001.pdf]

Cyclooxygenase-2 blockade can improve efficacy of VEGF-targeting drugs

Supplementary Material

Table S1: Primer sequences. Primers were purchased from Eurofins MWG as indicated.

Primers sequences

	**Forward primer**	**Reverse primer**
**murine *Ptgs2* (Cox-2)**	5'-GTCTTCCAGCCCATTGAACCT-3'	5'-GGAACACAGCTACGAAAACCC-3'
**murine *Tgfβ1***	5'-AAATCAACGGGATCAGCCCC-3'	5'-CGCACACAGCAGTTCTTCTC-3'
**murine *Fgf2***	5'-ACCCACACGTCAAACTACAACT-3'	5'-ACTGGAGTATTTCCGTGACCG-3'
**murine *Il-6***	5'-AGAAAGACAAAGCCAGAGTCCT-3'	5'-CTTGGTCCTTAGCCACTCCTT-3'
**murine *Vegf-a***	5'-TGCGGATCAAACCTCACCAA-3'	5'-TGTTCTGTCTTTCTTTGGTCTGC-3'
**murine *Hgf***	5'-GCCCTATTTCCCGTTGTGAAG-3'	5'-CCGCAGTTGTTTTGTTTTGGC-3'
**murine *Pdgf-d***	5'-CCCTCCAAGGATAACGTCAAGA-3'	5'-ACACCCCAGAGAAAGAGCTTGT-3'
**murine *Gapdh***	5'-TCAAGCTCATTTCCTGGTATGACA-3'	5'-CTCTTGCTCAGTGTCCTTGCTG-3'

**human *PTGS2* (Cox-2)**	5'-GTGCATTGGAATCAAGCCTGG-3'	5'-GGCAGAGTCCAAAGAAAGTGAAC-3'
**human *ACTA2* (α-SMA)**	5'-CGTTACTACTGCTGAGCGTGA-3'	5'-GATGGCTGGAACAGGGTCTC-3'
**human *FAP***	5'-GGGATGGTCATTGCCTTGGT-3'	5'-CTCCATAGGACCAGCCCCATA-3'
**human *GAPDH***	5'-AGGGCTGCTTTTAACTCTGGT-3'	5'-CCCCACTTGATTTTGGAGGGA-3'

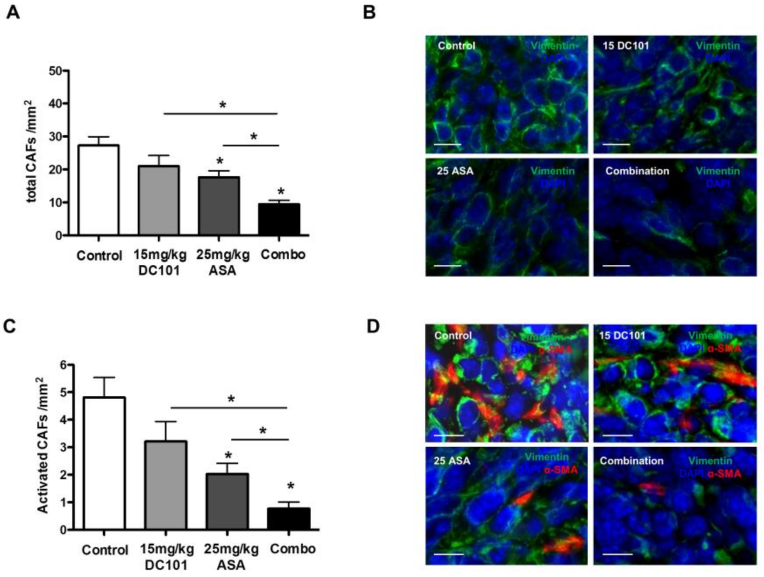

Supplemental Figure 1: DC101 and Cox-2 inhibition reduce the cancer-associated fibroblasts number and their activation. A-D, histomorphometric analyses of vimentin$^+$ total CAFs (A and B) and vimentin$^+$α-Sma$^+$ activated CAFs (C and D) in 4T1 tumor sections. Data indicates a reduction of total CAFs infiltration number with DC101 and ASA monotherapy and an additive effect with the combination (n = 7; $*P < 0.0001$) (A and B). Quantification of vimentin$^+$α-Sma$^+$ CAFs reveals a reduction in the fraction of activated CAFs upon ASA treatment (n = 7; $*P < 0.001$) (C and D). Representative pictures of immunofluorescence stained for vimentin (green), α-Sma (red) and DAPI in 4T1 tumor tissues (scale bar: 10 μm) (B and D).

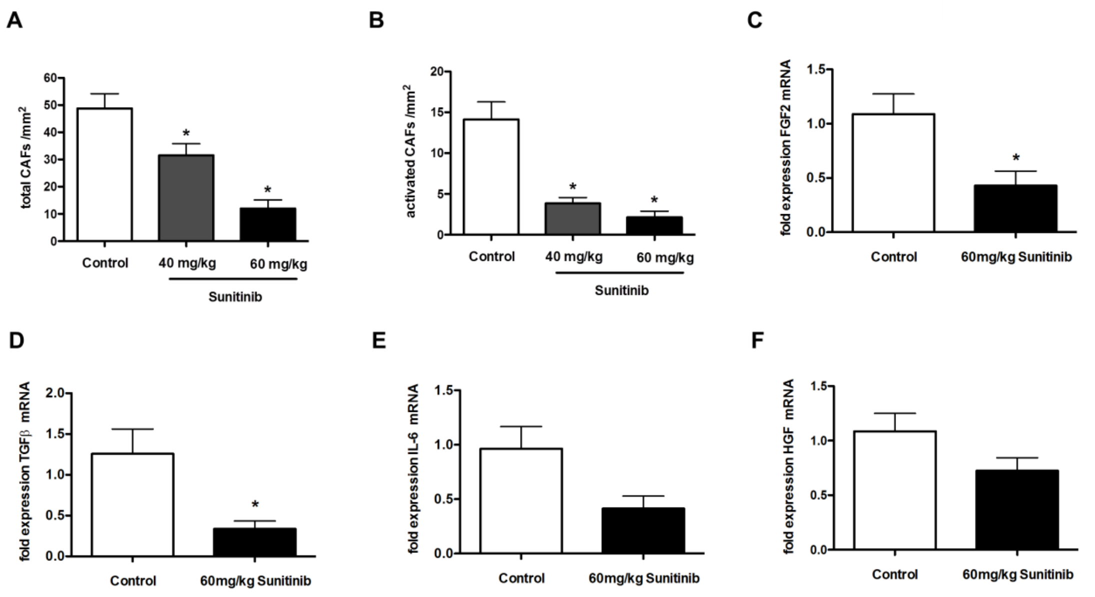

Supplemental Figure 2: High doses of sunitinib reduce tumor infiltration and activation of CAFs. A and B, histomorphometric quantification of vimentin$^+$ total CAFs (A) and vimentin$^+$α-Sma$^+$ activated CAFs (B) in 4T1 tumor sections. Data indicate a reduction of total CAF infiltration and activation with 40 and 60mg/kg sunitinib treatment, respectively (n = 7; *$P < 0.05$, *$P < 0.0001$).

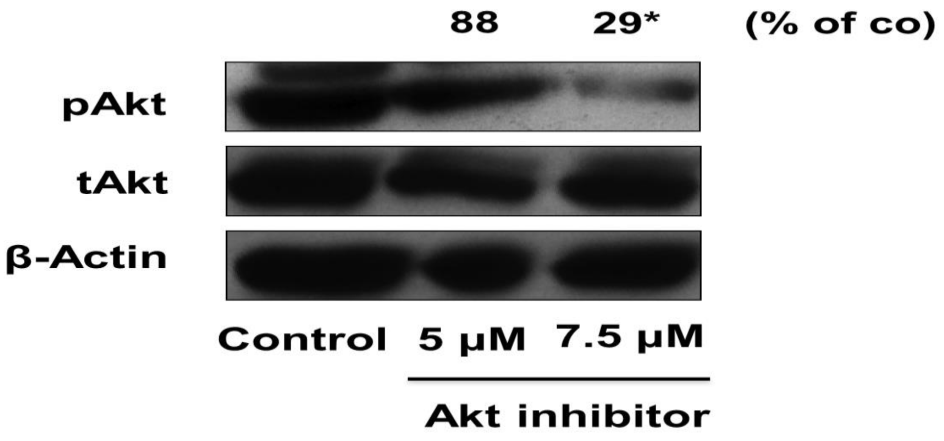

Supplemental Figure 3: Inhibition of Akt phosphorylation by MK-2206. Immunoblot showing protein levels of phosphorylated Akt (pAkt), total Akt (tAkt) and β-Actin from protein extracts of CAFs isolated from tumor tissue of n=2 lung cancer patients after 3 hours of incubation with 5 and 7.5 µM Akt inhibitor MK-2206, confirming the inhibition of Akt phosphylation after treatment. Densitometric quantification of (phosphorylated Akt/ β-Actin)/(total Akt/ β-Actin) (n = 3; *P = 0.0003)

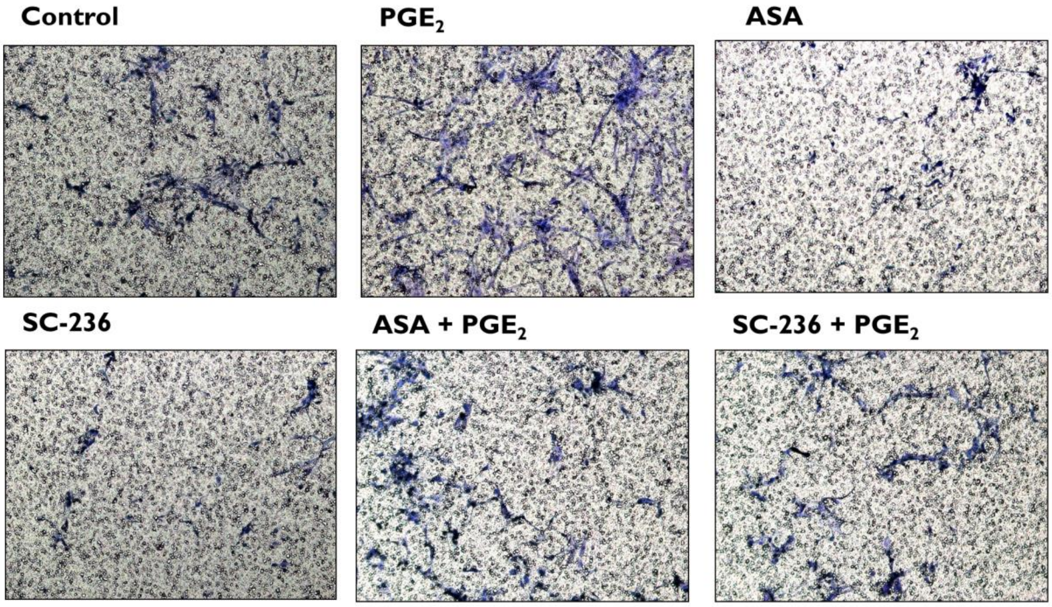

Supplemental Figure 4: Representative pictures of migration assay. It was performed with CAFs isolated from tumor tissue of n=2 lung cancer patients quantified in Figure 8B showing an increase in the migration ability by PGE_2 which is counteracted by ASA or SC-236 treatment ($n = 3$; $*P < 0.005$).

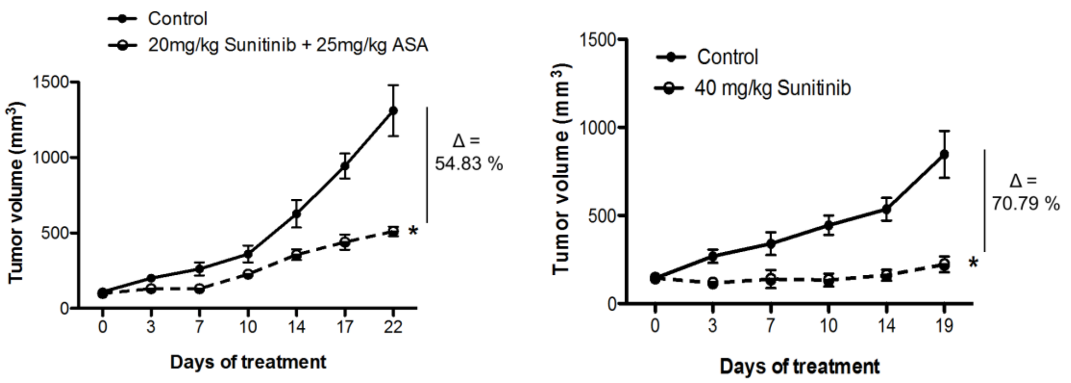

Supplemental Figure 5: 25 mg/kg ASA could substitute for a higher dose of sunitinib. Comparison between 4T1 tumor growth curves in mice treated with combination of 20mg/kg Sunitinib and 25mg/kg ASA or mice treated with 40mg/kg Sunitinib. It shows no significant difference in the tumor growth reduction (%) upon the high dose of sunitinib or the combinatory treatment. (Δ = tumor growth reduction expressed in %) (n = 6; P = 0.146).
